# Practitioner's Handbook of Diseases of the Ear and Naso-Pharynx

**Published:** 1887-12

**Authors:** 


*Practitioner's Handbook of Diseases of the Ear and Naso-Pharynx.* (Third Edition of the *Aural Surgery.*) By H. MACNAUGHTON JONES, M.D., M.CH., M.A.O. (Hon.) Pp. 176. London: J. & A. CHURCHILL. 1887.

This little work is written in the author's well-known agreeable style, and furnishes the reader with a good *resumé* of the best and latest work on the subjects treated of.

It is most profusely illustrated with woodcuts, and a special feature is the addition of coloured plates taken from the author's Aural Atlas, representing some of the more commonly occurring morbid states of the tympanum.

We are glad to see the author speaking strongly on the craze of some specialists for evulsion of the turbinated bones; and we are likewise quite at one with him in his denunciation of the use of instrumental interference in the case of foreign bodies in the meatus. "My belief is," he says, "from several years' experience, that syringing is *the* one safe and certain method of removing foreign bodies from the ear."

It would be well for a large number of poor children who come to our hospital casualty-rooms with beads and other foreign bodies in their ears, if these remarks were taken to heart by house-surgeons and dressers.

We recommend this book as one of the best of the numerous modern small works on Diseases of the Ear.

